# Does the Use of a Sanitary Sheath During Artificial Insemination Improve Pregnancy Outcomes in Cattle? A Systematic Review With Meta‐Analysis

**DOI:** 10.1111/rda.70325

**Published:** 2026-10-14

**Authors:** César Augusto Pinzón‐Osorio, Alondra González‐Patiño, Daniela Cortes‐Beltran

**Affiliations:** ^1^ College of Veterinary Medicine Universidade Federal de Pelotas (UFPEL) Pelotas Brazil; ^2^ Faculty of Veterinary Medicine and Animal Science Universidad Autónoma de Nuevo León Monterrey Mexico; ^3^ Department of Large Animal Medicine and Surgery, College of Veterinary Medicine University of Georgia Athens Georgia USA

**Keywords:** artificial insemination, cattle fertility, pregnancy per AI, protective AI covers, reproductive management

## Abstract

Sanitary sheaths (SS), used to reduce reproductive‐tract contamination during bovine artificial insemination (AI), have shown inconsistent associations with pregnancy per AI (P/AI). This systematic review with meta‐analysis evaluated whether SS use improves P/AI compared with AI without SS. The primary analysis comprised six independent cohorts contributing seven P/AI strata and 11,367 inseminations. Risk ratios (RRs) were pooled using random‐effects models with restricted maximum likelihood estimation and Hartung–Knapp adjustment. The use of SS did not significantly improve P/AI (RR = 1.07; 95% CI: 0.94–1.21; *p* = 0.245), although the point estimate corresponded to approximately 3.5 additional pregnancies per 100 inseminations. Heterogeneity was moderate (*I* ^2^ = 43.7%), and the 95% prediction interval crossed the null (RR 0.83–1.36), indicating that the effect may vary across production settings. Sensitivity analyses did not provide robust evidence of benefit: point estimates ranged from RR 1.07 to 1.10, statistical significance depended on dataset composition, and all prediction intervals crossed the null. Exploratory subgroup analyses showed no evidence that the effect differed by AI method, production system, or protective‐sheath type. Overall, current evidence does not demonstrate that SS use improves bovine P/AI and does not support its routine use as an effective fertility‐enhancing intervention; any potential benefit, if present, may be restricted to sanitary and management conditions that remain insufficiently characterised. Future adequately powered randomised trials are needed to determine whether reducing contamination through SS use causally improves fertility.

## 1 Introduction

Artificial insemination (AI) and timed artificial insemination (TAI) are fundamental tools for genetic improvement and reproductive management in cattle production systems worldwide (Baruselli et al. 2025; Monteiro et al. 2023). Pregnancy per AI (P/AI) reflects the combined effects of intrinsic animal‐related characteristics and extrinsic management, environmental, and procedural factors (Assis et al. 2025; López‐Gatius 2012; Pinzón‐Osorio et al. 2026; Ungerfeld et al. 2021). In recent years, hygienic strategies intended to limit reproductive tract contamination have received increasing attention because of their potential influence on fertility outcomes (Ault et al. 2019; Benner et al. 2018; Messman et al. 2020; Moreno et al. 2022).

One such strategy is the use of sanitary sheaths (SS), disposable barriers placed over the insemination catheter to minimise contact with the vulvar and vaginal environments before cervical passage, thereby potentially reducing microbial transfer to the cranial reproductive tract during AI (Bas et al. 2011; Hosie et al. 2019). This may be particularly relevant in TAI programs, where sequential AI under variable hygienic conditions, together with progesterone‐device‐associated changes in vaginal secretions and microbiota, may increase the risk of microbial transfer (Smith et al. 2024). Because the insemination catheter traverses the vagina and cervix before semen deposition, microorganisms encountered in the caudal reproductive tract may be mechanically transferred across the cervical barrier and introduced into the uterine environment (Bas et al. 2011). Among bacteria associated with bovine uterine disease, *Escherichia coli* and *Trueperella pyogenes* are particularly relevant, as their presence and persistence in the uterus have been associated with endometrial inflammation and impaired reproductive function (Carneiro et al. 2016; Sheldon et al. 2010). Such conditions may favour endometrial inflammatory responses (Salasel et al. 2010), including clinical or subclinical endometritis and, under more severe uterine disease conditions, pyometra (Sheldon et al. 2006, 2009). Persistent endometrial inflammation can impair uterine function and fertility, with subclinical endometritis being associated with reduced conception and an altered uterine environment capable of compromising embryo quality and survival (Hill and Gilbert 2008; Sheldon et al. 2009), thereby potentially impairing uterine receptivity and early pregnancy establishment (Ault et al. 2019; Zanella et al. 2024).

Sanitary sheath use has been described in commercial cattle operations, reproductive service programs, and technical recommendations (Cortés‐Beltrán and Gonella 2024; Panda et al. 2021; Walker 2020). However, despite its biological plausibility and practical adoption, whether limiting microbial transfer through SS use translates into improved P/AI remains uncertain. Individual studies have reported inconsistent findings, ranging from modest improvements in P/AI (Morales González et al. 2023) to no detectable effect on P/AI or non‐return outcomes across dairy and beef cattle populations (Kasimanickam 2016; King et al. 1984; Richards et al. 1984). Consequently, the use of SS appears to be supported more strongly by its hygienic rationale and field applicability than by consistent evidence of improved fertility. This synthesis of evidence therefore evaluated whether SS use during bovine AI, including TAI programs, improves P/AI compared with insemination performed without a sanitary sheath (NSS).

## 2 Methods

### 2.1 Research Strategy and Selection of Articles

This synthesis of evidence was conducted following the PRISMA (2020) guidelines. The study protocol was prospectively registered on the Open Science Framework (OSF; https://osf.io/8v63m). A comprehensive literature search was conducted in PubMed/MEDLINE, Scopus, Web of Science, CAB Abstracts, AGRIS, SciELO, and La Referencia. Database‐specific strategies were adapted following the approach of Jha et al. (2022), using combinations of keywords, Boolean operators, and terms related to cattle, artificial insemination, SS use, protective covers, contamination control, and pregnancy outcomes (Table S1). Reference lists of eligible studies and relevant review articles were also screened manually to identify additional records. No restrictions were imposed on year of publication. Studies published in English, Portuguese, or Spanish were considered eligible. Two reviewers independently screened records and assessed full texts, with disagreements resolved by consensus.

### 2.2 Eligibility Criteria and Data Extraction

Eligibility criteria were defined according to the PICOS framework (Table S2). Studies of dairy or beef cattle undergoing AI or TAI were eligible if they compared AI with SS versus without SS (NSS) and reported extractable group‐level P/AI or equivalent pregnancy or conception data. Non‐return outcomes were summarised narratively. Theses and conference reports were eligible when they provided original extractable data and sufficient methodological detail. Non‐bovine, non‐comparative, microbiological‐only, duplicate, and non‐original reports, as well as studies lacking a separable SS effect or calculable reproductive outcome, were excluded.

Two reviewers independently extracted study, population, insemination method, protective‐sheath type, pregnancy‐diagnosis, and group‐level outcome data using a standardised spreadsheet. Relevant animal‐, herd‐, semen‐, and management‐related variables were recorded when available. Independence of AI observations was assessed from study design and reporting; data not considered statistically independent at the analysed level were excluded from the primary meta‐analysis and retained for sensitivity analyses (Cumpston et al. 2019). Pregnancy counts were derived from reported percentages only when corresponding group denominators were available.

### 2.3 Risk of Bias Assessment

Risk of bias was assessed independently by two reviewers using SYRCLE's Risk of Bias tool for animal studies (Hooijmans et al. 2014), adapted to bovine AI experiments. Ten domains were judged to be at low, unclear, or high risk of bias based only on information explicitly reported in each article. Because blinding of inseminators to SS use was inherently infeasible, Domain 5 assessed whether treatment awareness could plausibly alter insemination technique, hygiene, handling, or other co‐interventions rather than operator blinding itself. Potential pseudoreplication and treatment‐by‐cluster confounding related to herd, lot, feeding, housing, repeated inseminations, or technician effects were also considered. Operational definitions and signalling questions for all domains are provided in Table S3.

### 2.4 Statistical Analysis

All analyses were performed in R (version 4.4.2; R Foundation for Statistical Computing, Vienna, Austria). Confirmed P/AI was synthesised as risk ratios (RRs) with 95% confidence intervals (CI), with RR > 1 favouring SS use. The primary analysis was restricted to data in which insemination observations were considered independent at the level analysed. An independent cohort was defined as an independent experimental population, whereas a stratum represented a separately reported SS‐versus‐control comparison within a cohort. Multiple eligible strata within a cohort were aggregated before meta‐analysis to preserve statistical independence by avoiding the treatment of correlated within‐cohort comparisons as independent effects and to prevent cohorts contributing multiple strata from exerting disproportionate influence on the pooled estimate. Cohort‐level RRs were calculated using the Mantel–Haenszel method and pooled under random‐effects models using restricted maximum likelihood estimation and Hartung–Knapp inference with an ad hoc standard‐error safeguard. Heterogeneity was assessed using Cochran's *Q*, *I* ^2^ (Borenstein 2023), *τ* ^2^, and 95% prediction intervals. Crude P/AI, model‐based absolute effects derived from the pooled RR and control risk, and random‐effects risk differences were also estimated.

Sensitivity and influence analyses evaluated alternative assumptions regarding data independence, overlapping reports, and uncertainties in extracted data using alternative dataset specifications, externally studentised residuals, influence diagnostics, Baujat plots, and leave‐one‐cohort‐out analyses. A multilevel model nested effects within cohorts and used CR2 cluster‐robust standard errors by cohort, with Satterthwaite degrees of freedom. Exploratory moderator analyses assessed production system, protective‐sheath type, and insemination method. Funnel plots were generated for the primary and dependence‐inclusive sensitivity datasets; Egger's, Begg's, and Harbord tests and trim‐and‐fill analysis were restricted to the primary independent dataset.

## 3 Results

### 3.1 Study Characteristics and Outcome Reporting

Database and supplementary searches identified 189 records. After removing duplicates, 66 records were screened, 12 full‐text articles were assessed, and one was excluded because it lacked an eligible comparator group. Eleven studies were included in the qualitative synthesis and 10 in the meta‐analysis (Figure 1). The P/AI evidence base comprised 10 studies published between 1984 and 2024, contributing 12 strata. Descriptive summaries were based on eight studies after accounting for overlapping evidence and unresolved data inconsistencies, comprising 22,005 inseminations. Studies were conducted across six countries. Dairy cattle accounted for 11,571 inseminations (52.6%) and beef cattle for 10,384 (47.2%); the production system was unreported for 50 inseminations (0.2%). Most observations involved cows (*n* = 21,415; 97.3%), followed by heifers (*n* = 540; 2.5%), with animal category unreported for 50 inseminations (0.2%). TAI represented 10,791 inseminations (49.0%), mixed TAI and estrus‐detected AI protocols 10,638 (48.3%), and estrus‐detected AI 576 (2.6%). Pregnancy was assessed by transrectal ultrasonography in 13,965 inseminations (63.5%), by either rectal palpation or ultrasonography in 7624 (34.6%), and by rectal palpation alone in 416 (1.9%). Of these eight studies, five (62.5%; *n* = 19,842 AI, 90.2%) evaluated flexible plastic bag‐type covers, whereas three (37.5%; *n* = 2163, 9.8%) evaluated rigid tube‐type protective sheaths. Detailed study characteristics are presented in Table 1. Bas et al. (2011) and Schuenemann et al. (2011) represented overlapping evidence, and Rezende et al. (2022) had unresolved data inconsistencies. In addition, the insemination observations analysed in Bas et al. (2011), Schuenemann et al. (2011), Hosie et al. (2019), and the dairy‐cattle stratum of Morales González et al. (2023) were not considered statistically independent at the analysed level. Consequently, the primary analysis comprised six independent cohorts and seven P/AI strata, representing 11,367 inseminations (5648 SS and 5719 NSS) and 6113 pregnancies. Crude P/AI was 55.3% with SS and 52.3% with NSS.

**FIGURE 1:**
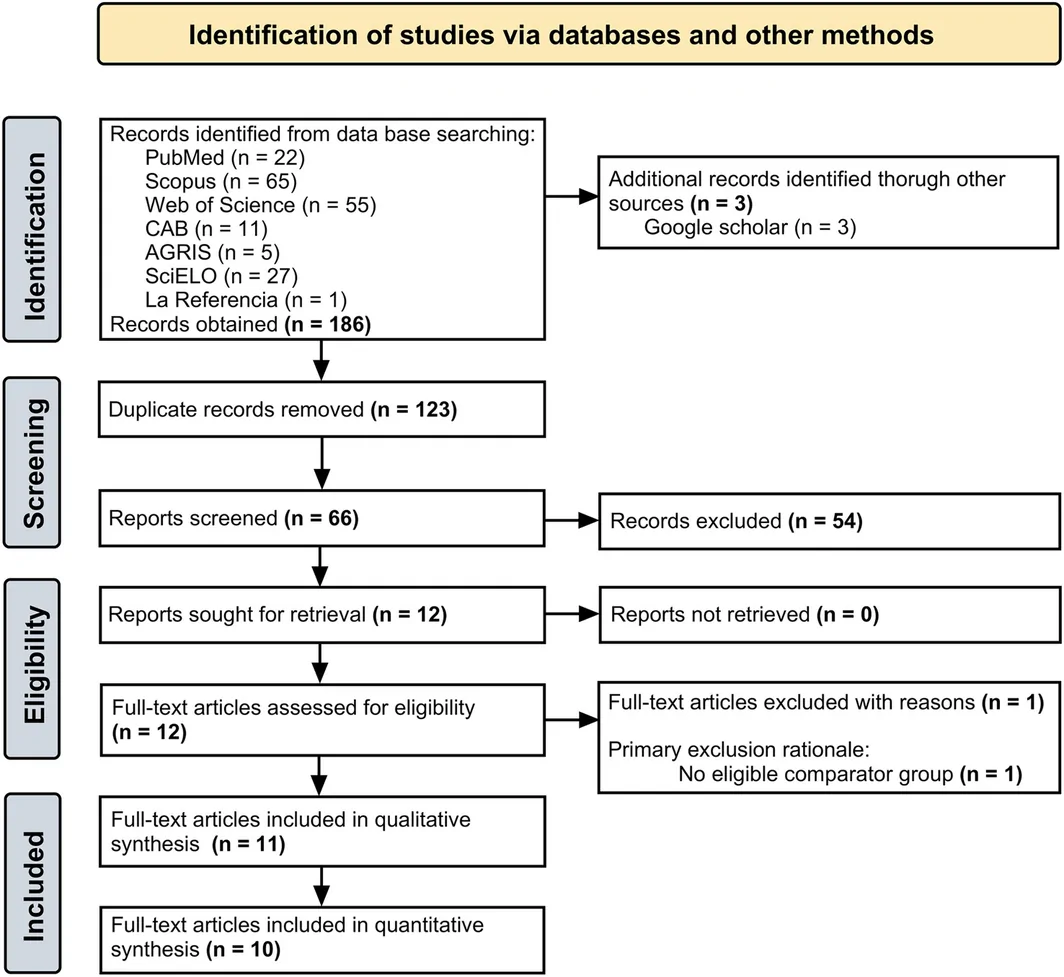
PRISMA 2020 flow diagram of study identification, screening, eligibility assessment, and inclusion. The single full‐text article excluded at the eligibility stage lacked an eligible comparator group.

**TABLE 1:** Characteristics and pregnancy outcomes of studies evaluating sanitary‐sheath use during bovine artificial insemination.

Study	Country	Production system	Animal category	SS type	AI method	Pregnancy diagnosis	P/AI with SS, n/N (%)	P/AI NSS, n/N (%)	Outcome	Primary analysis
King et al. (1984)	Canada	Dairy cattle	Cows	RT	Estrus‐detected AI	NRR	921/1485 (62.0)	1102/1783 (61.8)	NRR	No
King et al. (1984)	Canada	Dairy cattle	Cows	RT	Estrus‐detected AI	NRR	1072/1544 (69.4)	1047/1502 (69.7)	NRR	No
Richards et al. (1984)	USA	Dairy cattle	Cows	FPB	Estrus‐detected AI	NRR	3023/3876 (78.0)	2774/3511 (79.0)	NRR	No
Richards et al. (1984)	USA	Beef cattle	Cows	RT	Estrus‐detected AI	RP	116/203 (57.0)	132/213 (62.0)	P/AI	Yes
Schuenemann et al. (2011)^a^, ^b^	USA	Dairy cattle	Cows	RT	Mixed	TU	423/1405 (30.1)*	365/1438 (25.4)	P/AI	No
Bas et al. (2011)^a^, ^b^	USA	Dairy cattle	Cows	RT	Mixed	TU	208/487 (42.7)*	184/509 (36.1)	P/AI	No
Kasimanickam (2016)	USA	Beef cattle	Cows	RT	TAI	TU	95/160 (59.4)	90/161 (55.9)	P/AI	Yes
Kasimanickam (2016)	USA	Beef cattle	Heifers	RT	TAI	TU	127/218 (58.3)	121/212 (57.1)	P/AI	Yes
Silva et al. (2020)	Brazil	NR	NR	FPB	Estrus‐detected AI	TU	12/25 (48.0)	9/25 (36.0)	P/AI	Yes
Scariot et al. (2022)	Brazil	Dairy cattle	Cows	FPB	TAI	TU	164/374 (44.0)	152/449 (34.0)	P/AI	Yes
Rezende et al. (2022)^c^	Brazil	Beef cattle	Cows	FPB	TAI	TU	108/159 (67.9)	75/147 (51.0)	P/AI	No
Morales González et al. (2023)^a^	Uruguay	Dairy cattle	Cows	FPB	Mixed	TU	381/759 (50.2)	589/1259 (46.8)	P/AI	No
Morales González et al. (2023)	Uruguay	Beef cattle	Cows	FPB	TAI	TU	2578/4613 (55.9)*	2458/4604 (53.4)	P/AI	Yes
Hosie et al. (2019)^a^	Australia	Dairy cattle	Cows	FPB	Mixed	RP or TU	1345/3655 (36.8)	1441/3969 (36.3)	P/AI	No
Soytürk and Erdem (2024)	Türkiye	Dairy cattle	Heifers	FPB	Estrus‐detected AI	TU	31/55 (56.4)	28/55 (50.9)	P/AI	Yes

Abbreviations: AI, artificial insemination; FPB, flexible plastic bag‐type; NR, not reported; NRR, non‐return; NSS, no sanitary sheath; P/AI, pregnancy per artificial insemination; RP, rectal palpation; RT, rigid tube‐type; SS, sanitary sheath; TAI, timed artificial insemination; TU, transrectal ultrasonography.

^a^ Insemination observations were not considered statistically independent at the level analysed; therefore, these data were excluded from the independence‐restricted primary meta‐analysis and retained for sensitivity analyses.

^b^ Bas et al. (2011) and Schuenemann et al. (2011) represent overlapping evidence and were never included simultaneously in the same meta‐analytic specification.

^c^ Excluded from the primary analysis because of unresolved inconsistencies in the reported denominators/data and evaluated only in sensitivity analyses.

* Statistically significant SS–NSS difference (*p* < 0.05) for the corresponding extracted comparison.

### 3.2 Risk of Bias

Across the 11 reports, the main concerns were allocation procedures, incomplete reporting of outcome‐assessor blinding, and other design‐related sources of bias (Figure S1 and Table S3). No report was judged to be at low risk of bias for sequence generation or allocation concealment (Figure S1A,B): 36% used predictable allocation methods, whereas 64% did not describe how randomisation was generated. In 55% of reports, treatment groups were comparable for key reproductive factors such as parity, body condition, days postpartum, bull, service number, or synchronisation protocol, while 73% evaluated sanitary‐sheath and control inseminations concurrently under similar herd, feeding, housing, environmental, seasonal, and insemination‐management conditions. Because blinding of inseminators to SS use was inherently infeasible, performance bias was instead assessed according to whether treatment awareness could plausibly alter insemination technique, hygiene, handling, or other co‐interventions; 55% of reports were judged at low risk, 36% at unclear risk, and 9% at high risk for this domain. Blinding of pregnancy‐outcome assessors remained unclear in all reports, although pregnancy or non‐return outcomes were assessed using the same method and timing in 100% of reports (Figure S1A,B). Other potential sources of bias, including pseudoreplication, repeated inseminations, clustering, and management‐related confounding, were judged at low risk in 18%, unclear risk in 55%, and high risk in 27% of reports.

### 3.3 Effect of Sanitary Sheath Use on Pregnancy Per Artificial Insemination

Six independent cohorts contributed seven P/AI strata, comprising 11,367 inseminations: 5648 performed using SS and 5719 with NSS. In the primary analysis, SS use yielded a modest numerical increase in P/AI (RR = 1.07; 95% CI: 0.94–1.21; *p* = 0.245; Figure 2), equivalent to a 6.6% relative increase. Applying the pooled RR to the observed control P/AI (52.3%) gave a model‐based P/AI of 55.8% with SS, corresponding to an absolute difference of 3.49 percentage points. Heterogeneity was moderate (*I* ^2^ = 43.7%; *τ* ^2^ = 0.0067; *Q* = 8.88; *p* = 0.113), and the 95% prediction interval crossed the null (RR 0.83–1.36), which, using the observed control P/AI of 52.3% as reference, corresponds to approximately 43.4%–71.1% P/AI, or 9 fewer to 19 more pregnancies per 100 inseminations in a similar future setting.

**FIGURE 2:**
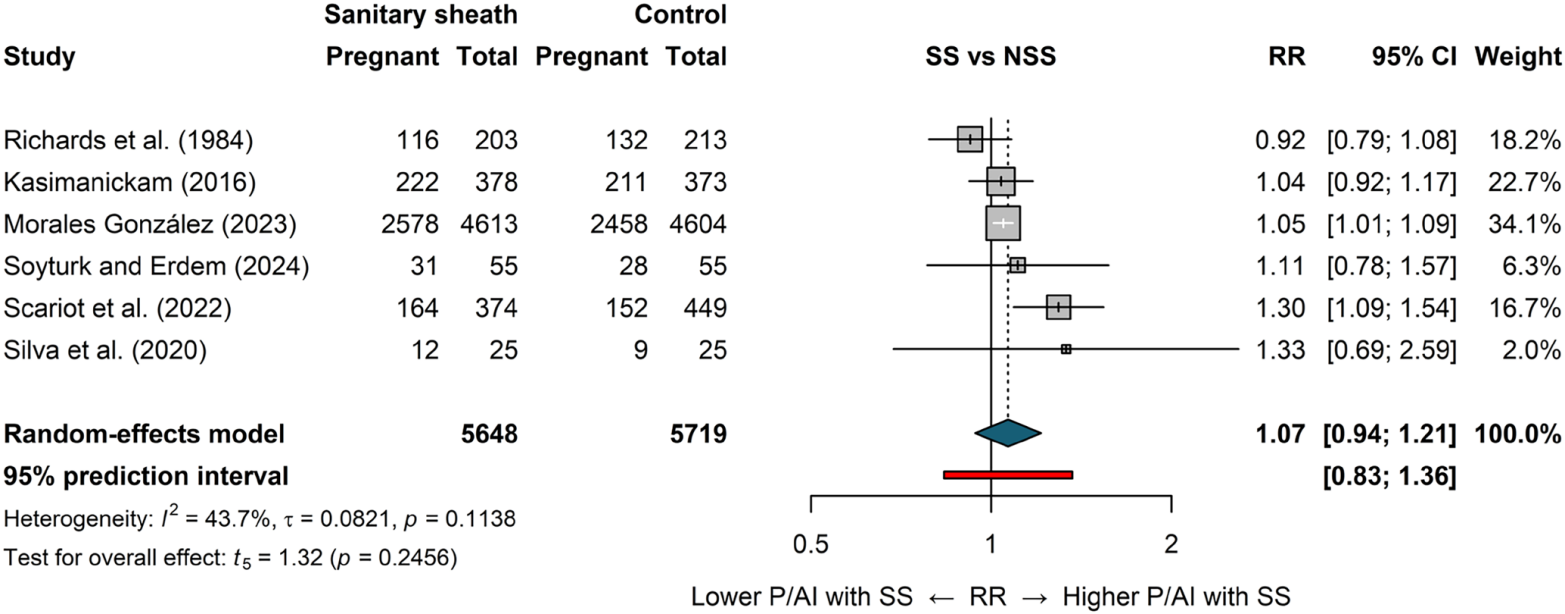
Forest plot comparing sanitary sheath (SS) versus no sanitary sheath (NSS) for pregnancy per artificial insemination (P/AI) in cattle. Risk ratios (RR) > 1 favour SS. The REML random‐effects model with Hartung–Knapp adjustment yielded an RR of 1.07 (95% CI: 0.94–1.21; *p* = 0.245), with moderate heterogeneity (*I* ^2^ = 43.7%; τ^2^ = 0.0067; *p* for heterogeneity = 0.113) and a 95% prediction interval of 0.83–1.36.

### 3.4 Sensitivity and Influence Analyses

When non‐independent AI data were additionally included (10,549 SS and 11,456 NSS AI), the analysis yielded a similar effect (RR = 1.07; 95% CI: 0.98–1.15; *p* = 0.102), with P/AI increasing from 45.43% to 48.40% (+2.97 percentage points). Sensitivity analyses consistently favoured SS use (RR 1.07–1.10); Sensitivity analyses A and B showed trends toward higher P/AI (*p* = 0.088 and 0.053, respectively), whereas Sensitivity analysis C reached nominal statistical significance (*p* = 0.048). All 95% prediction intervals crossed the null (Tables S4 and S5; Figures S2–S5). The pooled risk difference was 3.33 percentage points (95% CI: −2.08–8.75; *p* = 0.174), while the multilevel model reproduced the primary estimate (RR = 1.07; 95% CI: 0.94–1.21; *p* = 0.245; CR2 *p* = 0.257). Scariot et al. (2022) was potentially influential; its omission reduced heterogeneity to *I* ^2^ = 0% while preserving a favourable point estimate (RR = 1.04; 95% CI: 0.99–1.09; *p* = 0.084; Figure S6). Leave‐one‐cohort‐out estimates remained directionally consistent (RR 1.04–1.10), with no single cohort reversing the overall association.

### 3.5 Subgroup Analyses

Exploratory subgroup analyses provided no statistical evidence of effect modification by production system, protective‐sheath type, or AI method (all CR2 *p* ≥ 0.191; Table S6 and Figure S7). Although point estimates varied across subgroups, all 95% confidence and prediction intervals crossed the null. Interpretation was further limited for dairy cattle and rigid protective sheaths, each represented by only two independent cohorts.

### 3.6 Small‐Study Effects and Publication‐Bias Assessment

Exploratory analyses showed no statistical evidence of funnel‐plot asymmetry (Figure S8A), and trim‐and‐fill produced a virtually unchanged adjusted estimate (Egger: *t* = 0.5909, df = 4, *p* = 0.586; Begg: *z* = 1.3151, *p* = 0.188; Harbord: *t* = 0.5919, df = 4, *p* = 0.585; RR = 1.06; 95% CI: 0.97–1.16) after imputing one potentially missing comparison (Figure S8B). However, with only six independent cohorts, these analyses had limited ability to detect or exclude publication bias or other small‐study effects. A separate funnel plot including the prespecified non‐independent AI data was examined descriptively but was not used for primary inference (Figure S8C). Influence diagnostics identified Scariot et al. (2022) as potentially influential, with the greatest joint contribution to heterogeneity and influence on the pooled estimate (Figure S9A,B).

## 4 Discussion

To our knowledge, this is the first systematic review and quantitative synthesis to estimate the reproductive effect of SS use during bovine AI. A central implication of our findings is that an apparent hygienic benefit does not necessarily translate into improved reproductive outcomes. In the primary analysis, SS use yielded a small numerical increase in P/AI, corresponding to a model‐based estimate of approximately 3.5 additional pregnancies per 100 inseminations. Sensitivity analyses produced consistently favourable point estimates, equivalent to approximately 3.0–4.3 additional pregnancies per 100 inseminations, depending on the analytic specification. However, statistical support for the association was limited; the primary random‐effects estimate was inconclusive, between‐cohort heterogeneity was moderate, and the 95% prediction interval ranged from RR 0.83 to 1.36, indicating that effects of SS use across comparable settings could vary from a modest reduction to a potentially meaningful improvement in P/AI. Notably, reintroducing the prespecified data with non‐independent AI observations produced a similar pooled estimate (RR = 1.07), but with narrower confidence and prediction intervals, indicating that restricting the primary analysis to independent AI data primarily reduced precision rather than materially altering the estimated effect magnitude. Across the primary and sensitivity analyses, all prediction intervals crossed the null. This variability may reflect differences in production systems, animal categories, insemination protocols, pregnancy diagnosis methods, and baseline hygiene conditions. Overall, the evidence supports a small but uncertain average effect rather than a consistent fertility benefit across production settings, and does not support routine SS adoption solely to improve P/AI.

Bas et al. (2011) showed that SS reduced bacterial recovery from catheter tips after AI, confirming their function as a mechanical hygienic barrier; however, this finding demonstrates reduced catheter‐tip contamination rather than reduced uterine exposure or improved fertility. The pathway linking lower catheter contamination to reduced intrauterine bacterial burden, attenuated endometrial inflammation, improved embryo survival, and higher P/AI remains unproven. This distinction is biologically relevant because the inflammatory response after insemination may be transient and physiological rather than inherently pathological. Bull sperm induce a transient endometrial proinflammatory response, whereas excessive or persistent polymorphonuclear‐cell infiltration has been associated with reduced conception (Kaufmann et al. 2009; Marey et al. 2023). Consistent with a magnitude‐ and persistence‐dependent effect, cytological endometritis detected at AI has been associated with reduced pregnancy outcomes in dairy cows and heifers (Pascottini et al. 2016, 2017). The reproductive consequence of microbial exposure may also depend on the physiological environment at insemination. Classic experimental work indicated greater uterine resistance to an intrauterine bacterial challenge at estrus than during the luteal phase, with subsequent evidence of cycle‐dependent variation in neutrophil activity and endometrial responses to bacterial stimuli (Ault et al. 2019; Chacin et al. 1990; Hussain and Daniel 1991; Rowson et al. 1953). Thus, the oestrogen‐dominated peri‐estrous environment may partly limit the consequences of low‐level microbial exposure (Brownlie and Hibbitt 1972; Rowson et al. 1953), reducing the scope for SS to improve fertility when baseline contamination is low. Interestingly, Bas et al. (2011) reported no SS‐associated improvement at first service but higher P/AI with SS at second or subsequent services, a pattern compatible with, but insufficient to establish a greater role for hygiene after repeated inseminations. Importantly, pregnancy outcomes at later services may also reflect selection of cows that remained nonpregnant after previous inseminations, reproductive diagnosis, treatment of underlying disorders, hormonal interventions, and changes in uterine or ovarian status between services. Therefore, the higher P/AI observed with SS at second or subsequent services cannot be attributed to SS alone when these potential co‐interventions and time‐varying reproductive factors are not adequately accounted for.

Contemporary reproductive‐tract microbiome studies further support the concept that microbial exposure is not uniformly detrimental to fertility. Ault et al. (2019) identified differences in uterine bacterial‐community structure shortly before TAI between cows that subsequently became pregnant and those that did not, whereas other studies found no overall pregnancy‐associated differences in vaginal diversity (Messman et al. 2020), very low uterine microbial biomass after AI in heifers (Moraes et al. 2024), or altered uterine communities without a consistent relationship with pregnancy (Figueiredo et al. 2024). Collectively, these findings indicate that the reproductive consequences of microbial exposure depend on its anatomical location, abundance, composition, timing, and interaction with uterine health and host inflammatory responses, rather than on microbial detection alone. Accordingly, SS use would be expected to improve P/AI only when it prevents biologically meaningful uterine exposure, rather than merely reducing detectable contamination. Current evidence does not establish a well‐defined quantitative bacterial‐load threshold associated with impaired pregnancy outcomes (Bicalho et al. 2017; Miranda‐CasoLuengo et al. 2019; Santos et al. 2011). Operationally, biologically meaningful exposure could be characterised by increased uterine bacterial burden and/or enrichment of taxa associated with uterine dysbiosis or disease, including *Fusobacterium*, *Bacteroides*, *Prevotella*, and *Porphyromonas*, particularly when accompanied by evidence of endometrial inflammation (Poole 2026).

Against this biological background, SS may be better regarded as a conditional hygienic safeguard rather than a direct fertility‐enhancing technology. Its reproductive value is likely to depend on the magnitude and biological relevance of the microbial challenge prevented and on the capacity of the reproductive tract to contain the resulting inflammatory response. In this context, SS could improve fertility primarily when microbial exposure exceeds the normal defensive capacity of the reproductive tract, such as under poor perineal hygiene, contaminated facilities, repeated catheter manipulation, inconsistent vulvar hygiene, suboptimal insemination technique, or compromised uterine health. Because these conditions were inconsistently reported, their role in determining whether SS use translates into improved P/AI remains biologically plausible but unproven. In anestrous dairy cows, post‐treatment vaginitis scores were unrelated to pregnancy at first AI or other reproductive outcomes (Walsh et al. 2007), whereas in beef cattle undergoing TAI, neither the severity of CIDR‐associated vaginal discharge nor visible secretion on the AI sheath was associated with P/AI outcomes (Dias et al. 2019). Thus, visible contamination or discharge may be an inadequate proxy for the biologically relevant uterine microbial challenge that SS is intended to prevent. Exploratory analyses provided no statistical evidence that the association differed by production system, protective‐sheath type, or AI method. However, moderator inference was limited by the small number of independent cohorts, particularly for dairy cattle and rigid protective sheaths.

From an implementation perspective, even a small absolute improvement could be relevant in large breeding programs if the effect is reproducible and the marginal value of an additional pregnancy exceeds the total cost of SS use. At the primary point estimate, the model‐based absolute difference was 3.49 percentage points, corresponding descriptively to approximately one additional pregnancy per 29 inseminations with SS. However, the potential value of SS should not be considered solely in terms of additional pregnancies. As a hygienic barrier, SS may provide ancillary benefits by reducing microbial contamination during AI and, if this translates into lower uterine exposure, could potentially reduce the risk of uterine inflammation or infection and their associated reproductive and treatment costs. These potential benefits remain to be directly demonstrated and quantified, as the available studies primarily evaluated pregnancy outcomes rather than uterine health or economic endpoints. The estimated fertility effect should be interpreted cautiously, as the confidence and prediction intervals remained compatible with no benefit. Its economic value therefore remains uncertain and will depend on the reproducibility of the fertility effect, baseline risk, and the incremental costs of SS implementation. Because SS are single‐use plastic devices, broader adoption would also increase plastic waste, underscoring the need for lower‐impact or biodegradable alternatives in bovine production systems (Borreani and Tabacco 2017; FAO 2021). Nevertheless, if the reproductive and broader hygienic benefits of SS are confirmed, these benefits may offset the additional implementation costs and environmental burden associated with their use. Establishing this balance will require studies that jointly evaluate reproductive performance, uterine health, economic outcomes, and environmental impacts.

The present evidence base has several limitations. Only six independent cohorts contributed seven P/AI strata; several reports contained non‐independent AI observations, and incomplete reporting of allocation and blinding procedures limited assessment of potential bias. Restricting the primary synthesis to independent observations preserved the pooled effect magnitude but reduced precision, while the small number of cohorts limited evaluation of biologically relevant effect modifiers.

Future randomised trials should compare standardised vulvar hygiene alone with hygiene plus SS, prospectively define the experimental unit, and account for repeated inseminations, herd clustering, and technician effects. These studies should also evaluate SS across clearly defined reproductive‐management settings, including AI following detected natural estrus and synchronisation protocols with or without intravaginal progesterone devices, to determine whether the reproductive context modifies any potential effect of SS on fertility. Animals or herds should be prospectively stratified according to baseline contamination risk using standardised measures of perineal cleanliness, vulvar preparation, environmental hygiene, and vaginal or vestibular bacterial burden. Future studies should also examine whether SS provides an additional biosecurity benefit by limiting contamination of insemination equipment and potential mechanical transfer of reproductive pathogens, including *Campylobacter fetus* subsp. *venerealis* and *Tritrichomonas foetus*, where epidemiologically relevant (Depner 2017; Truyers et al. 2014). Simultaneous assessment of catheter‐tip and reproductive‐tract bacterial load, equipment contamination, endometrial cytology or inflammatory biomarkers, confirmed pregnancy, and cost‐effectiveness would clarify the causal pathway and permit formal evaluation of whether baseline contamination modifies the reproductive response to SS.

## 5 Conclusion

This evidence synthesis does not demonstrate that SS use improves P/AI in cattle. Although the pooled point estimate was directionally favourable, substantial statistical uncertainty and the limited evidence base do not support considering SS an effective fertility‐enhancing intervention. Any reproductive benefit, if present, may depend on sanitary or management conditions that remain insufficiently defined. Accordingly, SS should be regarded primarily as a hygienic measure rather than a routine fertility‐enhancing intervention until adequately powered trials determine whether reproductive benefits exist under specific conditions.

## Author Contributions

C.A.P.O.: conceptualisation, methodology, formal analysis, investigation, validation, writing original draft – review and editing. A.G.P: methodology, formal analysis, writing – review and editing. D.C.B: methodology, writing original draft – review and editing.

## Funding

The authors are grateful for funding provided by the Coordenação de Aperfeiçoamento de Pessoal de Nível Superior (CAPES/Brazil; Financial Code 001), Conselho Nacional de Desenvolvimento Científico e Tecnológico (CNPq), and Fundação de Amparo à Pesquisa no Estado do Rio Grande do Sul (FAPERGS).

## Ethics Statement

The authors have nothing to report.

## Conflicts of Interest

The authors declare no conflicts of interest.

## Supporting information


**Table S1:** Search strategy used in PubMed/MEDLINE, Scopus, Web of Science, CAB Abstracts, AGRIS, SciELO, Google Scholar and La Referencia databases to identify studies evaluating the effect of sanitary sheath or equivalent protective covers used during artificial insemination on reproductive outcomes in cattle. Searches were last updated on July, 7, 2026.
**Table S2:** Eligibility criteria for study selection, based on the PICOS framework (Population, Intervention, Comparator, Outcome, and Study Design).
**Table S3:** Primary and sensitivity random‐effects meta‐analyses comparing confirmed pregnancy per artificial insemination between sanitary‐sheath (SS) and control inseminations (NSS) in cattle.
**Table S3A:** Relative‐effect estimates.
**Table S3B:** Absolute‐scale interpretation.
**Table S4:** Additional sensitivity analyses of the association between sanitary‐sheath use and confirmed pregnancy per artificial insemination.
**Table S5:** Exploratory subgroup analyses of sanitary‐sheath (SS) use and confirmed pregnancy per artificial insemination (P/AI) in cattle.
**Table S5A:** Relative‐effect estimates.
**Table S5B:** Absolute‐scale interpretation by subgroup.
**Table S6:** Operational definitions and signalling questions used to assess risk of bias across the 10 adapted SYRCLE domains in studies evaluating sanitary‐sheath use during bovine artificial insemination.
**Figure S1:** Risk‐of‐bias assessment of studies evaluating sanitary‐sheath (SS) use during bovine artificial insemination. (A) Distribution of low, unclear, and high risk‐of‐bias judgements across the 11 reports for each of the 10 domains of the adapted SYRCLE tool. Percentages represent the proportion of reports assigned to each judgement category within a given domain. (B) Study‐level traffic‐light plot showing the judgement for each report and domain. Green circles with “+” indicate low risk of bias, amber circles with “?” indicate unclear risk of bias, and red circles with “−” indicate high risk of bias. Each report contributed one judgement per domain.
**Figure S2:** Sensitivity analysis of the aggregated random‐effects meta‐analysis evaluating sanitary sheath (SS) use versus insemination without a sanitary sheath (NSS) for pregnancy per artificial insemination (P/AI) after reintroducing the prespecified data with non‐independent insemination observations. Eight cohorts contributed 22,005 inseminations (10,549 SS and 11,456 NSS). Squares represent cohort‐specific risk ratios (RRs), with size proportional to random‐effects weight, and horizontal lines indicate 95% confidence intervals (CIs). The pooled random‐effects estimate was RR = 1.07 (95% CI: 0.98–1.15; *p* = 0.102), with moderate heterogeneity (*I* ^2^ = 44.8%; *τ* = 0.0576; *p* for heterogeneity = 0.080) and a 95% prediction interval of 0.91–1.25. The vertical solid line denotes the null effect (RR = 1), the dotted line the pooled estimate, the diamond the pooled RR and 95% CI, and the red bar the 95% prediction interval.
**Figure S3:** Sensitivity A random‐effects meta‐analysis evaluating sanitary sheath (SS) use versus insemination without a sanitary sheath (NSS) for pregnancy per artificial insemination (P/AI) in cattle, with Schuenemann et al. (2011) replacing the overlapping Bas et al. (2011) report and Rezende et al. (2022) excluded. Eight cohorts contributed 23,852 inseminations (11,467 SS and 12,385 NSS). Squares represent cohort‐specific risk ratios (RRs), with size proportional to random‐effects weight, and horizontal lines indicate 95% confidence intervals (CIs). The pooled random‐effects estimate was RR = 1.07 (95% CI: 0.99–1.17; *p* = 0.088), with moderate heterogeneity (*I* ^2^ = 51.1%; *τ* = 0.0686; *p* for heterogeneity = 0.045) and a 95% prediction interval of 0.89–1.29. The vertical solid line denotes the null effect (RR = 1), the dotted line the pooled estimate, the diamond the pooled RR and 95% CI, and the red bar the 95% prediction interval.
**Figure S4:** Sensitivity B random‐effects meta‐analysis evaluating sanitary sheath (SS) use versus insemination without a sanitary sheath (NSS) for pregnancy per artificial insemination (P/AI) in cattle, with Bas et al. (2011) retained, Rezende et al. (2022) included, and the overlapping Schuenemann et al. (2011) report excluded. Nine cohorts contributed 22,311 inseminations (10,708 SS and 11,603 NSS). Squares represent cohort‐specific risk ratios (RRs), with size proportional to random‐effects weight, and horizontal lines indicate 95% confidence intervals (CIs). The pooled random‐effects estimate was RR = 1.10 (95% CI: 1.00–1.20; *p* = 0.053), with moderate heterogeneity (*I* ^2^ = 56.3%; *τ* = 0.0885; *p* for heterogeneity = 0.019) and a 95% prediction interval of 0.88–1.37. The vertical solid line denotes the null effect (RR = 1), the dotted line the pooled estimate, the diamond the pooled RR and 95% CI, and the red bar the 95% prediction interval.
**Figure S5:** Sensitivity C random‐effects meta‐analysis evaluating sanitary sheath (SS) use versus insemination without a sanitary sheath (NSS) for pregnancy per artificial insemination (P/AI) in cattle, with Schuenemann et al. (2011) replacing the overlapping Bas et al. (2011) report and Rezende et al. (2022) included. Nine cohorts contributed 24,158 inseminations (11,626 SS and 12,532 NSS). Squares represent cohort‐specific risk ratios (RRs), with size proportional to random‐effects weight, and horizontal lines indicate 95% confidence intervals (CIs). The pooled random‐effects estimate was RR = 1.10 (95% CI: 1.00–1.20; *p* = 0.048), with moderate heterogeneity (*I* ^2^ = 59.7%; *τ* = 0.0900; *p* for heterogeneity = 0.011) and a 95% prediction interval of 0.87–1.38. The vertical solid line denotes the null effect (RR = 1), the dotted line the pooled estimate, the diamond the pooled RR and 95% CI, and the red bar the 95% prediction interval.
**Figure S6:** Leave‐one‐independent‐cohort‐out sensitivity analysis of the aggregated random‐effects meta‐analysis evaluating sanitary sheath (SS) use versus insemination without a sanitary sheath (NSS) for pregnancy per artificial insemination (P/AI) in cattle. Each row shows the pooled risk ratio (RR) after sequentially omitting one independent cohort. The primary random‐effects estimate was RR = 1.07 (95% CI: 0.94–1.21; *p* = 0.245), with a 95% prediction interval of 0.83–1.36. All leave‐one‐cohort‐out point estimates remained above the null, ranging from RR = 1.04 to 1.10. However, all corresponding confidence intervals included 1.00, with *p*‐values ranging from 0.008 to 0.342. Residual heterogeneity ranged from *I* ^2^ = 0% to 54.8% across iterations. Omission of Scariot et al. (2022) produced the greatest reduction in heterogeneity (*I* ^2^ = 0%; *τ* ^2^ = 0), indicating that this cohort contributed importantly to between‐cohort variability.
**Figure S7:** Forest plots of exploratory subgroup analyses of sanitary‐sheath (SS) use versus insemination without a sanitary sheath (NSS) for confirmed pregnancy per artificial insemination (P/AI) in cattle. (A) Artificial‐insemination method (estrus‐detected AI vs. TAI). (B) Production system (beef vs. dairy cattle). C. Protective‐sheath type (flexible plastic bag‐type cover vs. rigid tube‐type protective sheath). Points represent subgroup‐specific pooled RRs, black horizontal lines indicate 95% confidence intervals, and red horizontal lines indicate 95% prediction intervals. The vertical line denotes the null effect (RR = 1). Differences between subgroup levels were evaluated using CR2 cluster‐robust inference with Satterthwaite small‐sample degrees of freedom.
**Figure S8:** Funnel‐plot and small‐study‐effect sensitivity analyses for sanitary‐sheath (SS) use and pregnancy per artificial insemination (P/AI) in cattle. (A) Contour‐enhanced funnel plot for the independence‐restricted primary meta‐analysis, including six independent cohorts. (B) Trim‐and‐fill analysis of the primary independent dataset, showing the observed cohorts and one potentially missing comparison imputed on the left, together with the original and adjusted pooled estimates. In all panels, the *x*‐axis represents the log risk ratio and the *y*‐axis the corresponding standard error. Shaded regions indicate two‐sided significance contours of *p* < 0.10, *p* < 0.05, and *p* < 0.01 relative to the null effect. Funnel‐plot asymmetry and trim‐and‐fill analyses were considered exploratory because of the limited number of independent cohorts. (C) Descriptive funnel plot after reintroducing the prespecified non‐independent AI data; Schuenemann et al. (2011), representing an overlapping report, and Rezende et al. (2022), with unresolved data inconsistencies, are overlaid for transparency but were not included in this sensitivity model.
**Figure S9:** Influence diagnostics for the primary random‐effects meta‐analysis of sanitary‐sheath (SS) use and pregnancy per artificial insemination (P/AI). (A) Study‐level influence diagnostics derived from the random‐effects model, including externally studentised residuals (*rstudent*), DFFITS, Cook's distance, covariance ratios, residual heterogeneity after study deletion (*τ* ^2^.del and QE.del), leverage values (*hat*), and random‐effects weights. Horizontal reference lines indicate the diagnostic thresholds generated by the influence‐analysis procedure. Scariot et al. (2022) had a studentised residual of 2.8149, exceeding the Bonferroni‐adjusted threshold of 2.638, and was flagged as potentially influential. (B) Baujat plot showing each study's contribution to overall heterogeneity on the *x*‐axis and its influence on the pooled estimate on the *y*‐axis. Scariot et al. (2022) occupied the upper‐right region, indicating the largest joint contribution to heterogeneity and influence. Study numbers correspond to: 1, Richards et al. (1984); 2, Kasimanickam (2016); 3, Silva et al. (2020); 4, Scariot et al. (2022); 5, Morales González et al. (2023); and 6, Soytürk and Erdem (2024).

## Data Availability

The data that support the findings of this study are available from the corresponding author upon reasonable request.
