# Supplementary material for: Does the Use of a Sanitary Sheath During Artificial Insemination Improve Pregnancy Outcomes in Cattle? A Systematic Review With Meta‐Analysis
Source: Reprod Domest Anim. 2026 Oct 14;61(10):e70325. doi: 10.1111/rda.70325 (PMC13664662; doi:10.1111/rda.70325)
Supplement: Supplementary file 1 [file RDA-61-e70325-s001.docx]

**Supplementary data**

**Supplementary Table S1.** Search strategy used in PubMed/MEDLINE, Scopus, Web of Science, CAB Abstracts, AGRIS, SciELO, Google Scholar and La Referencia databases to identify studies evaluating the effect of sanitary sheath or equivalent protective covers used during artificial insemination on reproductive outcomes in cattle. Searches were last updated on July 7, 2026.

PubMed

(cattle[MeSH Terms] OR "Cattle"[Title/Abstract] OR bovine[Title/Abstract] OR cattle[Title/Abstract] OR cow*[Title/Abstract] OR heifer*[Title/Abstract] OR "dairy cow*"[Title/Abstract] OR "beef cow*"[Title/Abstract] OR "Bos taurus"[Title/Abstract] OR "Bos indicus"[Title/Abstract] OR zebu[Title/Abstract] OR crossbred*[Title/Abstract]) AND ("Artificial Insemination"[MeSH Terms] OR "artificial insemination"[Title/Abstract] OR inseminat*[Title/Abstract] OR "timed artificial insemination"[Title/Abstract] OR TAI[Title/Abstract] OR "fixed-time artificial insemination"[Title/Abstract] OR "fixed time artificial insemination"[Title/Abstract] OR FTAI[Title/Abstract] OR IATF[Title/Abstract] OR "estrus synchronization"[Title/Abstract] OR "oestrus synchronization"[Title/Abstract] OR synchronization[Title/Abstract] OR synchronisation[Title/Abstract] OR estrus[Title/Abstract] OR oestrus[Title/Abstract]) AND ("sanitary sheath"[Title/Abstract] OR "sanitary sheaths"[Title/Abstract] OR "protective sheath"[Title/Abstract] OR "protective sheaths"[Title/Abstract] OR "double sheath"[Title/Abstract] OR "double sheaths"[Title/Abstract] OR "AI gun cover"[Title/Abstract] OR "insemination gun cover"[Title/Abstract] OR "protective cover"[Title/Abstract] OR "sanitary cover"[Title/Abstract] OR "catheter cover"[Title/Abstract] OR "insemination sheath"[Title/Abstract] OR "AI sheath"[Title/Abstract] OR "vaginal contamination"[Title/Abstract] OR "uterine contamination"[Title/Abstract] OR "bacterial contamination"[Title/Abstract] OR contamination[Title/Abstract]) AND (pregnancy[Title/Abstract] OR pregnant[Title/Abstract] OR conception[Title/Abstract] OR fertility[Title/Abstract] OR "pregnancy rate"[Title/Abstract] OR "conception rate"[Title/Abstract] OR "pregnancy per artificial insemination"[Title/Abstract] OR "pregnancy per AI"[Title/Abstract] OR P/AI[Title/Abstract] OR "non-return rate"[Title/Abstract] OR "non return rate"[Title/Abstract] OR "return to estrus"[Title/Abstract] OR "return to oestrus"[Title/Abstract])

Scopus

TITLE-ABS-KEY (bovine OR cattle OR cow* OR heifer* OR "dairy cow*" OR "beef cow*" OR "Bos taurus" OR "Bos indicus" OR zebu OR crossbred*) AND TITLE-ABS-KEY ("artificial insemination" OR inseminat* OR "timed artificial insemination" OR TAI OR "fixed-time artificial insemination" OR "fixed time artificial insemination" OR FTAI OR IATF OR "estrus synchronization" OR "oestrus synchronization" OR synchronization OR synchronisation OR estrus OR oestrus) AND TITLE-ABS-KEY ("sanitary sheath" OR "sanitary sheaths" OR "protective sheath" OR "protective sheaths" OR "double sheath" OR "double sheaths" OR "AI gun cover" OR "insemination gun cover" OR "protective cover" OR "sanitary cover" OR "catheter cover" OR "insemination sheath" OR "AI sheath" OR "vaginal contamination" OR "uterine contamination" OR "bacterial contamination" OR contamination) AND TITLE-ABS-KEY (pregnancy OR pregnant OR conception OR fertility OR "pregnancy rate" OR "conception rate" OR "pregnancy per artificial insemination" OR "pregnancy per AI" OR "non-return rate" OR "non return rate" OR "return to estrus" OR "return to oestrus")

Web of Science

TS = ((bovine OR cattle OR cow* OR heifer* OR "dairy cow*" OR "beef cow*" OR "Bos taurus" OR "Bos indicus" OR zebu OR crossbred*) AND ("artificial insemination" OR inseminat* OR "timed artificial insemination" OR TAI OR "fixed-time artificial insemination" OR "fixed time artificial insemination" OR FTAI OR IATF OR "estrus synchronization" OR "oestrus synchronization" OR synchronization OR synchronisation OR estrus OR oestrus) AND ("sanitary sheath" OR "sanitary sheaths" OR "protective sheath" OR "protective sheaths" OR "double sheath" OR "double sheaths" OR "AI gun cover" OR "insemination gun cover" OR "protective cover" OR "sanitary cover" OR "catheter cover" OR "insemination sheath" OR "AI sheath" OR "vaginal contamination" OR "uterine contamination" OR "bacterial contamination" OR contamination) AND (pregnancy OR pregnant OR conception OR fertility OR "pregnancy rate" OR "conception rate" OR "pregnancy per artificial insemination" OR "pregnancy per AI" OR "non-return rate" OR "non return rate" OR "return to estrus" OR "return to oestrus"))

CAB Abstracts

(cattle OR bovin* OR cow* OR heifer* OR "dairy cow*" OR "beef cow*" OR "Bos taurus" OR "Bos indicus" OR zebu* OR nelore OR crossbred*) AND ("artificial insemination" OR inseminat* OR "timed artificial insemination" OR TAI OR FTAI OR IATF OR "fixed-time artificial insemination" OR "fixed time artificial insemination" OR "estrus synchronization" OR "oestrus synchronization" OR synchronization OR synchronisation OR "estrus detection" OR "oestrus detection" OR estrus OR oestrus) AND ("sanitary sheath" OR "sanitary sheaths" OR "protective sheath" OR "protective sheaths" OR "protective plastic sheath" OR "protective plastic sheaths" OR "protective AI cover sheath" OR "protective AI cover sheaths" OR "plastic cover sheath" OR "plastic cover sheaths" OR "cover sheath" OR "cover sheaths" OR "sheath protector" OR "sheath protectors" OR "disposable sheath cover" OR "disposable sheath covers" OR "sanitary cover" OR "sanitary covers" OR "sanitary sleeve" OR "sanitary sleeves" OR "Sani-Shield" OR "Sani Shield" OR "double sheath" OR "double sheaths" OR "double-sheath" OR "double sheath insemination" OR "double sheath breeding" OR "AI gun cover" OR "AI gun covers" OR "insemination gun cover" OR "insemination gun covers" OR "artificial insemination gun cover" OR "insemination sheath cover" OR "catheter cover" OR "AI gun contamination" OR "insemination gun contamination" OR "vaginal contamination" OR "uterine contamination" OR "reproductive tract contamination")

AGRIS (International System for Agricultural Science and Technology)

(cattle OR bovine OR bovines OR cow OR cows OR heifer OR heifers OR "dairy cow" OR "dairy cows" OR "beef cow" OR "beef cows" OR "Bos taurus" OR "Bos indicus" OR zebu OR nelore OR crossbred OR crossbreds OR bovino OR bovinos OR bovina OR bovinas OR vaca OR vacas OR novilha OR novilhas OR novillo OR novillos OR vaquillona OR vaquillonas OR "ganado bovino" OR "gado bovino") AND ("artificial insemination" OR insemination OR inseminations OR "timed artificial insemination" OR TAI OR FTAI OR IATF OR "fixed-time artificial insemination" OR "fixed time artificial insemination" OR "estrus synchronization" OR "oestrus synchronization" OR "estrus detection" OR "oestrus detection" OR "inseminación artificial" OR "inseminacion artificial" OR "inseminación a tiempo fijo" OR "inseminacion a tiempo fijo" OR "sincronización del estro" OR "sincronizacion del estro" OR "inseminação artificial" OR "inseminacao artificial" OR "inseminação artificial em tempo fixo" OR "inseminacao artificial em tempo fixo" OR "sincronização do estro" OR "sincronizacao do estro") AND ("sanitary sheath" OR "sanitary sheaths" OR "protective sheath" OR "protective sheaths" OR "protective plastic sheath" OR "protective plastic sheaths" OR "protective AI cover sheath" OR "protective AI cover sheaths" OR "plastic cover sheath" OR "plastic cover sheaths" OR "cover sheath" OR "cover sheaths" OR "sheath protector" OR "sheath protectors" OR "disposable sheath cover" OR "sanitary cover" OR "sanitary sleeve" OR "sanitary sleeves" OR "Sani-Shield" OR "Sani Shield" OR "double sheath" OR "double sheaths" OR "double-sheath" OR "double sheath insemination" OR "double sheath breeding" OR "AI gun cover" OR "insemination gun cover" OR "artificial insemination gun cover" OR "insemination sheath cover" OR "catheter cover" OR "vaginal contamination" OR "uterine contamination" OR "AI gun contamination" OR "insemination gun contamination" OR "bainha sanitária" OR "bainha sanitaria" OR "bainha protetora" OR "capa sanitária" OR "camisa sanitaria" OR "capa sanitaria" OR "capa protetora" OR "dupla bainha" OR "funda sanitaria" OR "vaina sanitaria" OR "funda protectora" OR "vaina protectora" OR "doble funda" OR "doble vaina" OR "contaminación vaginal" OR "contaminacion vaginal" OR "contaminación uterina" OR "contaminacion uterina" OR "contaminação vaginal" OR "contaminacao vaginal" OR "contaminação uterina" OR "contaminacao uterina")

SciELO

(cattle OR bovine OR bovines OR cow OR cows OR heifer OR heifers OR "Bos taurus" OR "Bos indicus" OR zebu OR nelore OR bovino OR bovinos OR bovina OR bovinas OR vaca OR vacas OR novilha OR novilhas OR novillo OR novillos OR vaquillona OR vaquillonas) AND ("artificial insemination" OR insemination OR inseminations OR "timed artificial insemination" OR TAI OR FTAI OR IATF OR "fixed-time artificial insemination" OR "fixed time artificial insemination" OR "inseminación artificial" OR "inseminacion artificial" OR "inseminación a tiempo fijo" OR "inseminacion a tiempo fijo" OR "inseminação artificial" OR "inseminacao artificial" OR "inseminação artificial em tempo fixo" OR "inseminacao artificial em tempo fixo") AND ("sanitary sheath" OR "sanitary sheaths" OR "protective sheath" OR "protective sheaths" OR "protective plastic sheath" OR "protective plastic sheaths" OR "plastic cover sheath" OR "plastic cover sheaths" OR "cover sheath" OR "cover sheaths" OR "sheath protector" OR "sheath protectors" OR "double sheath" OR "double sheaths" OR "AI gun cover" OR "insemination gun cover" OR "sanitary cover" OR "sanitary sleeve" OR "vaginal contamination" OR "uterine contamination" OR "bainha sanitária" OR "bainha sanitaria" OR "bainha protetora" OR "bainhas protetoras" OR "capa sanitária" OR "capa sanitaria" OR "capa protetora" OR "capas protetoras" OR "dupla bainha" OR "duplo revestimento" OR "contaminação vaginal" OR "contaminacao vaginal" OR "contaminação uterina" OR "contaminacao uterina" OR "funda sanitaria" OR "fundas sanitarias" OR "vaina sanitaria" OR "vainas sanitarias" OR "funda protectora" OR "fundas protectoras" OR "doble funda" OR "doble vaina" OR "contaminación vaginal" OR "contaminacion vaginal" OR "contaminación uterina" OR "contaminacion uterina")

La Referencia

"sanitary sheath" AND cattle AND "artificial insemination"

"protective plastic sheath" AND cattle AND insemination

"protective AI cover sheath" AND cattle

"sheath protector" AND bovine AND insemination

"double sheath" AND cattle AND insemination

"AI gun cover" AND cattle

"insemination gun cover" AND bovine

"vaginal contamination" AND cattle AND "artificial insemination"

"bainha sanitária" AND bovinos AND "inseminação artificial"

"bainha protetora" AND bovinos AND "inseminação artificial"

"capa protetora" AND bovinos AND "inseminação artificial"

"dupla bainha" AND bovinos AND inseminação

"contaminação vaginal" AND bovinos AND "inseminação artificial"

"funda sanitaria" AND bovinos AND "inseminación artificial"

"funda protectora" AND bovinos AND "inseminación artificial"

"camisa sanitaria" AND bovinos AND "inseminación artificial"

"doble funda" AND bovinos AND inseminación

"contaminación vaginal" AND bovinos AND "inseminación artificial"

**Supplementary Table S2.** Eligibility criteria for study selection, based on the PICOS framework (Population, Intervention, Comparator, Outcome, and Study Design).

| Parameter | Inclusion criterion | Exclusion criterion |
| --- | --- | --- |
| Population (P) | Studies involving female cattle, including Bos taurus, Bos indicus, zebu, dairy cattle, beef cattle, crossbred cattle, cows, or heifers subjected to artificial insemination. Eligible populations may include nulliparous, primiparous, or multiparous females inseminated after detected estrus or under timed artificial insemination (TAI) protocols. Studies involving both dairy and beef animals were eligible when reproductive outcomes could be extracted for cattle receiving AI or TAI. | Studies involving species other than cattle or studies not involving in vivo AI/TAI in females. Studies focused exclusively on bulls, semen collection, semen processing, in vitro fertilization, embryo transfer, cloning, superovulation, oocyte recovery, or laboratory semen/microbial outcomes without female fertility data were excluded from the primary synthesis. |
| Intervention (I) | Use of a sanitary sheath, protective cover, protective sleeve, or disposable barrier placed over the insemination catheter, AI gun, or applicator during bovine AI or TAI to reduce contact between the insemination device and the vaginal or cervical environment before semen deposition. Eligible studies may include SS use in estrus-based AI, synchronized AI, or TAI programs, regardless of cattle category, production system, or hormonal protocol. | Studies evaluating hygiene or reproductive management practices without a clearly defined SS intervention. Studies assessing only vulvar cleaning, perineal hygiene, antiseptic washing, lubricant use, semen type, hormonal protocol, insemination timing, antibiotic treatment, or catheter type without separable SS use were excluded. |
| Comparator (C) | Artificial insemination performed without a sanitary sheath or protective cover, under otherwise comparable reproductive management conditions. Eligible comparators include conventional AI or TAI using the same or similar semen type, insemination system, synchronization protocol, animal category, and management context, provided that the control group did not receive SS. | Studies without a no-SS control group. Studies comparing different brands, materials, or techniques of SS use without a non-SS control group. Studies in which the comparator differed simultaneously in major reproductive interventions, such as semen type, hormonal protocol, insemination timing, antibiotic therapy, or animal category, preventing isolation of the SS effect. |
| Outcome (O) | Primary eligible outcomes were reproductive fertility endpoints after AI or TAI, including pregnancy per artificial insemination (P/AI), pregnancy rate, conception rate, or confirmed pregnancy diagnosis. Eligible studies had to provide extractable numerical data, such as number of females inseminated and number pregnant/conceived in each group, or sufficient information to derive an effect size. Non-return to estrus was considered an indirect fertility proxy and was summarized narratively when reported. | Studies that did not report pregnancy, conception, fertility, or non-return outcomes after AI or TAI. Studies reporting only bacterial contamination, vaginal or uterine microbiota, cytology, inflammation, endometritis, behavioral responses, or procedural contamination without a reproductive endpoint were excluded from the primary meta-analysis. Studies with incomplete or non-extractable outcome data were excluded unless additional data could be obtained from authors or reliable supplementary sources. |
| Study Design (S) | Controlled experimental studies, randomized or non-randomized field trials, factorial studies, prospective cohort studies, retrospective cohort studies, or commercial field studies comparing AI or TAI performed with SS versus without SS. Theses, technical reports, and conference proceedings may be screened for additional references or included only if they provide original extractable data and meet all eligibility criteria. | Expert opinions, narrative reviews without original data, book chapters, manuals, producer guidelines, editorials, commentaries, case reports, conference abstracts without sufficient numerical data, duplicate publications, and studies with insufficient methodological detail to determine population, intervention, comparator, or reproductive outcome. |

**Table S3.** Operational definitions and signaling questions used to assess risk of bias across the 10 adapted SYRCLE domains in studies evaluating sanitary-sheath use during bovine artificial insemination.

| Domain | Recommended signaling question |
| --- | --- |
| D1. Sequence generation | Was the allocation sequence adequately generated to assign females or insemination units to sanitary sheath (SS) or control treatment? |
| D2. Baseline characteristics | Were the SS and control groups comparable at baseline with respect to key animal, reproductive, semen, and management factors that could influence fertility? |
| D3. Allocation concealment | Was treatment allocation adequately concealed before females or insemination units were assigned to SS or control? |
| D4. Comparable management/environment | Were SS and control animals managed under comparable herd, lot, feeding, housing, environmental, seasonal, and AI/TAI conditions? |
| D5. Performance bias related to treatment awareness | Given that blinding of inseminators to SS use was inherently infeasible, was the risk that knowledge of treatment allocation altered insemination technique, hygiene, handling, or other co-interventions adequately minimized? |
| D6. Standardized outcome assessment | Was pregnancy or conception assessed using the same method, timing, and procedures in the SS and control groups, independently of treatment allocation? |
| D7. Blinding of outcome assessment | Were pregnancy-outcome assessors blinded to SS/control allocation, or was outcome ascertainment sufficiently objective and standardized that knowledge of treatment assignment was unlikely to influence the assessment? |
| D8. Incomplete outcome data | Were exclusions, losses to follow-up, missing pregnancy diagnoses, and changes in denominators adequately reported and balanced between SS and control groups? |
| D9. Selective outcome reporting | Were all prespecified or expected fertility outcomes, treatment comparisons, and relevant subgroup results reported completely and consistently with the stated methods? |
| D10. Other sources of bias | Were other important sources of bias unlikely, including inappropriate experimental units, pseudoreplication, repeated inseminations, shared controls, herd or technician clustering, and confounding by semen, sire, inseminator, protocol, animal category, hygiene, or other management factors? |

**Abbreviations:** AI, artificial insemination; SS, sanitary sheath; TAI, timed artificial insemination. Risk-of-bias judgments were classified as low, unclear, or high based on information explicitly reported in each study. Blinding of inseminators was considered inherently infeasible because SS use was directly observable during AI; therefore, Domain 5 assessed the potential for treatment awareness to influence insemination technique, hygiene, handling, or other co-interventions rather than operator blinding itself.

**Table S4.** Primary and sensitivity random-effects meta-analyses comparing confirmed pregnancy per artificial insemination between sanitary-sheath (SS) and control inseminations (NSS) in cattle

**Table S4A.** Relative-effect estimates

| Analysis specification | Effects | Cohorts | AI, SS | AI, NSS | RR | 95% CI | SE log RR | *p* | I² (%) | τ² | Q (df) | *p* Q | 95% PI |
| --- | --- | --- | --- | --- | --- | --- | --- | --- | --- | --- | --- | --- | --- |
| Primary: independent AI data only | 7 | 6 | 5,648 | 5,719 | 1.07 | 0.94–1.21 | 0.04 | 0.245 | 43.7 | 0.006 | 8.883 (5) | 0.113 | 0.83–1.36 |
| Sensitivity: primary + non-independent AI data | 10 | 8 | 10,549 | 11,456 | 1.07 | 0.98–1.15 | 0.03 | 0.102 | 44.8 | 0.003 | 12.676 (7) | 0.080 | 0.91–1.25 |
| Sensitivity A: Schuenemann et al. (2011) replaces Bas et al. (2011); Rezende et al. (2022) excluded | 10 | 8 | 11,467 | 12,385 | 1.07 | 0.99–1.17 | 0.03 | 0.088 | 51.1 | 0.004 | 14.321 (7) | 0.045 | 0.89–1.29 |
| Sensitivity B: Bas et al. (2011) retained; Rezende et al. (2022) included | 11 | 9 | 10,708 | 11,603 | 1.10 | 1.00–1.20 | 0.04 | 0.053 | 56.3 | 0.007 | 18.308 (8) | 0.019 | 0.88–1.37 |
| Sensitivity C: Schuenemann et al. (2011) replaces Bas et al. (2011); Rezende et al. (2022) included | 11 | 9 | 11,626 | 12,532 | 1.10 | 1.00–1.20 | 0.04 | 0.048 | 59.7 | 0.008 | 19.830 (8) | 0.011 | 0.87–1.38 |

**Abbreviations:** CI = confidence interval; RR = risk ratio; SE = standard error; SS = sanitary sheath; NSS = no sanitary sheath; τ² = estimated between-study variance.

Random-effects models were fitted using restricted maximum likelihood estimation with Hartung–Knapp adjustment and an ad hoc standard-error safeguard. Prediction intervals were calculated using the *t* distribution. The primary analysis was restricted to data in which AI observations were considered independent at the analyzed level. Accordingly, Bas et al. (2011), Schuenemann et al. (2011), Hosie et al. (2019), and the dairy-cattle stratum of Morales González (2023) were excluded from the primary analysis because of non-independent AI observations; the beef-cattle stratum of Morales González (2023) remained eligible. Sensitivity A replaced Bas et al. (2011) with Schuenemann et al. (2011) within the dependence-inclusive sensitivity specification. Sensitivity B added Rezende et al. (2022) to the dependence-inclusive specification retaining Bas et al. (2011). Sensitivity C replaced Bas et al. (2011) with Schuenemann et al. (2011) and included Rezende et al. (2022) within the dependence-inclusive specification. Bas et al. (2011) and Schuenemann et al. (2011) were never included simultaneously.

**Table S4B.** Absolute-scale interpretation

| Analysis | Crude P/AI, SS (%) | Crude P/AI, NSS (%) | Model-based P/AI: NSS → SS, Δ pp | RD (pp) | 95% CI RD | *p* RD |
| --- | --- | --- | --- | --- | --- | --- |
| Primary independent | 55.29 | 52.28 | 52.28 → 55.78, +3.49 | 3.33 | −2.08 to 8.75 | 0.174 |
| Primary + non-independent AI data | 47.94 | 45.43 | 45.43 → 48.40, +2.97 | 3.12 | −0.25 to 6.49 | 0.064 |
| Sensitivity A | 45.98 | 43.48 | 43.48 → 46.65, +3.17 | 2.98 | 0.05 to 5.91 | 0.047 |
| Sensitivity B | 48.24 | 45.50 | 45.50 → 49.82, +4.33 | 4.31 | −0.02 to 8.65 | 0.051 |
| Sensitivity C | 46.28 | 43.57 | 43.57 → 47.84, +4.27 | 3.95 | −0.04 to 7.95 | 0.051 |

**Abbreviations:** CI = confidence interval; P/AI = pregnancy per artificial insemination; RD = risk difference; SS = sanitary sheath; NSS = no sanitary sheath; Δ pp = absolute difference in percentage points.

† Observed control P/AI was calculated by dividing the total number of pregnancies by the total number of inseminations in the control groups under each specification. Model-based P/AI with SS was estimated by multiplying the corresponding control P/AI by the pooled RR. The resulting Δ pp values are translations of the RR to the absolute scale and are distinct from the independently pooled random-effects RD estimates.

**Table S5.** Additional sensitivity analyses of the association between sanitary-sheath use and confirmed pregnancy per artificial insemination

| Analysis | Effects | Cohorts | Effect estimate | 95% CI | *p* | 95% PI | Heterogeneity / variance | Interpretation |
| --- | --- | --- | --- | --- | --- | --- | --- | --- |
| Primary cohort-level RR model | 6 | 6 | RR = 1.07 | 0.94–1.21 | 0.245 | 0.83–1.36 | τ² = 0.0067; τ = 0.0821; I² = 43.7% | Positive average point estimate, but statistically inconclusive |
| Primary effect-level multilevel model | 7 | 6 | RR = 1.07 | 0.94–1.21 | 0.245 | 0.83–1.36 | σ² between cohorts = 0.0067; σ² within cohorts ≈ 0 | Estimate essentially identical to the cohort-level model |
| Multilevel model with CR2 robust inference | 7 | 6 | RR = 1.07 | —‡ | 0.257 | —‡ | Robust SE = 0.0475; Satterthwaite df = 3.35 | Inference remained inconclusive after robust variance correction |
| Primary risk-difference model | 6 | 6 | RD = 3.33 pp | −2.08 to 8.75 pp | 0.174 | −6.44 to 13.11 pp | τ² = 0.0010; I² = 34.4%; Q = 7.6277, P = 0.177 | Positive average absolute effect with substantial uncertainty |
| Leave-one-cohort-out analysis | 6 per iteration | 6 per iteration | RR = 1.04–1.10 | All CIs included 1.00 | 0.084–0.342 | 0.88–1.34 | I² = 0–54.8% | Direction remained positive, but precision and heterogeneity were sensitive to individual cohorts |

**Abbreviations:** AI = artificial insemination; CI = confidence interval; CR2 = bias-reduced cluster-robust variance estimator; P/AI = pregnancy per artificial insemination; RD = risk difference; RR = risk ratio; SE = standard error; σ² = multilevel variance component; τ² = between-study variance.

The multilevel model retained all seven P/AI strata and specified effects as nested within independent cohorts. Robust inference used CR2 standard errors clustered by cohort with Satterthwaite small-sample degrees of freedom. The estimated within-cohort between-effect variance approached zero, indicating that retaining the separate strata did not materially alter the pooled estimate.

‡ The displayed multilevel confidence and prediction intervals were obtained from the model-based multilevel estimator. Because the current CR2 output provided the robust standard error, degrees of freedom, and *P*-value but did not directly print a CR2 confidence interval or prediction interval, model-based and robust inferential quantities were not combined in the same row.

**Table S6.** Exploratory subgroup analyses of sanitary-sheath (SS) use and confirmed pregnancy per artificial insemination (P/AI) in cattle

**Table S6A.** Relative-effect estimates

| Moderator | Subgroup | Effects | Cohorts | AI, SS | AI, NSS | RR | 95% CI | 95% PI | *p* for subgroup difference† |
| --- | --- | --- | --- | --- | --- | --- | --- | --- | --- |
| AI method | Estrus-detected AI | 3 | 3 | 283 | 293 | 1 | 0.76–1.29 | 0.70–1.40 | 0.449 |
|  | TAI | 4 | 3 | 5,365 | 5,426 | 1.1 | 0.94–1.28 | 0.83–1.44 | — |
| Production system | Beef cattle | 4 | 3 | 5,194 | 5,190 | 1 | 0.98–1.10 | 0.98–1.10 | 0.191 |
|  | Dairy cattle | 2 | 2 | 429 | 504 | 1.3 | 0.98–1.61 | 0.98–1.61 | — |
| Protective-sheath type | Flexible plastic bag-type cover | 4 | 4 | 5,067 | 5,133 | 1.1 | 0.94–1.37 | 0.83–1.54 | 0.283 |
|  | Rigid tube-type protective sheath | 3 | 2 | 581 | 586 | 1 | 0.79–1.23 | 0.70–1.37 | — |

**Abbreviations:** AI = artificial insemination; CI = confidence interval; NSS = no sanitary sheath; PI = prediction interval; RR = risk ratio; SS = sanitary sheath; TAI = timed artificial insemination.

† *P* values correspond to the CR2 cluster-robust test of the difference between subgroup levels, with Satterthwaite small-sample degrees of freedom. They do not represent tests of the pooled effect within either individual subgroup.

**Table S6B.** Absolute-scale interpretation by subgroup

| **Moderator** | **Subgroup** | **Crude P/AI, SS (%)** | **Crude P/AI, NSS (%)** | **Model-based P/AI: NSS → SS (%)** | **Δ pp** |
| --- | --- | --- | --- | --- | --- |
| AI method | Estrus-detected AI | 56.18 | 57.68 | 57.68 → 57.11 | −0.57 |
|  | TAI | 55.25 | 51.99 | 51.99 → 56.96 | +4.97 |
| Production system | Beef cattle | 56.14 | 53.97 | 53.97 → 56.11 | +2.15 |
|  | Dairy cattle | 45.45 | 35.71 | 35.71 → 44.85 | +9.14 |
| Protective-sheath type | Flexible plastic bag-type cover | 54.96 | 51.57 | 51.57 → 58.35 | +6.78 |
|  | Rigid tube-type protective sheath | 58.18 | 58.53 | 58.53 → 57.61 | −0.92 |

**Abbreviations:** AI = artificial insemination; NSS = no sanitary sheath; P/AI = pregnancy per artificial insemination; SS = sanitary sheath; TAI = timed artificial insemination; Δ pp = model-based absolute difference in percentage points.

† Crude SS and NSS P/AI values represent the observed event proportions within the data contributing to each subgroup and are not meta-analytically pooled proportions. Model-based P/AI with SS was calculated by applying the subgroup-specific pooled RR to the corresponding observed NSS baseline P/AI. Accordingly, Δ pp is an absolute-scale translation of the RR and should not be interpreted as an independently pooled risk difference.

**FIGURE S1.** Risk-of-bias assessment of studies evaluating sanitary-sheath (SS) use during bovine artificial insemination. **A.** Distribution of low, unclear, and high risk-of-bias judgments across the 11 reports for each of the 10 domains of the adapted SYRCLE tool. Percentages represent the proportion of reports assigned to each judgment category within a given domain. **B.** Study-level traffic-light plot showing the judgment for each report and domain. Green circles with “+” indicate low risk of bias, amber circles with “?” indicate unclear risk of bias, and red circles with “−” indicate high risk of bias. Each report contributed one judgment per domain.

**FIGURE S2.** Sensitivity analysis of the aggregated random-effects meta-analysis evaluating sanitary sheath (SS) use versus insemination without a sanitary sheath (NSS) for pregnancy per artificial insemination (P/AI) after reintroducing the prespecified data with non-independent insemination observations. Eight cohorts contributed 22,005 inseminations (10,549 SS and 11,456 NSS). Squares represent cohort-specific risk ratios (RRs), with size proportional to random-effects weight, and horizontal lines indicate 95% confidence intervals (CIs). The pooled random-effects estimate was RR = 1.07 (95% CI: 0.98–1.15; *p*=0.102), with moderate heterogeneity (I² = 44.8%; τ = 0.0576; *p* for heterogeneity = 0.080) and a 95% prediction interval of 0.91–1.25. The vertical solid line denotes the null effect (RR = 1), the dotted line the pooled estimate, the diamond the pooled RR and 95% CI, and the red bar the 95% prediction interval.

**FIGURE S3.** Sensitivity A random-effects meta-analysis evaluating sanitary sheath (SS) use versus insemination without a sanitary sheath (NSS) for pregnancy per artificial insemination (P/AI) in cattle, with Schuenemann et al. (2011) replacing the overlapping Bas et al. (2011) report and Rezende et al. (2022) excluded. Eight cohorts contributed 23,852 inseminations (11,467 SS and 12,385 NSS). Squares represent cohort-specific risk ratios (RRs), with size proportional to random-effects weight, and horizontal lines indicate 95% confidence intervals (CIs). The pooled random-effects estimate was RR = 1.07 (95% CI: 0.99–1.17; *p*=0.088), with moderate heterogeneity (I² = 51.1%; τ = 0.0686; *p* for heterogeneity = 0.045) and a 95% prediction interval of 0.89–1.29. The vertical solid line denotes the null effect (RR = 1), the dotted line the pooled estimate, the diamond the pooled RR and 95% CI, and the red bar the 95% prediction interval.

**FIGURE S4.** Sensitivity B random-effects meta-analysis evaluating sanitary sheath (SS) use versus insemination without a sanitary sheath (NSS) for pregnancy per artificial insemination (P/AI) in cattle, with Bas et al. (2011) retained, Rezende et al. (2022) included, and the overlapping Schuenemann et al. (2011) report excluded. Nine cohorts contributed 22,311 inseminations (10,708 SS and 11,603 NSS). Squares represent cohort-specific risk ratios (RRs), with size proportional to random-effects weight, and horizontal lines indicate 95% confidence intervals (CIs). The pooled random-effects estimate was RR = 1.10 (95% CI: 1.00–1.20; *p*=0.053), with moderate heterogeneity (I² = 56.3%; τ = 0.0885; *p* for heterogeneity = 0.019) and a 95% prediction interval of 0.88–1.37. The vertical solid line denotes the null effect (RR = 1), the dotted line the pooled estimate, the diamond the pooled RR and 95% CI, and the red bar the 95% prediction interval.

**FIGURE S5.** Sensitivity C random-effects meta-analysis evaluating sanitary sheath (SS) use versus insemination without a sanitary sheath (NSS) for pregnancy per artificial insemination (P/AI) in cattle, with Schuenemann et al. (2011) replacing the overlapping Bas et al. (2011) report and Rezende et al. (2022) included. Nine cohorts contributed 24,158 inseminations (11,626 SS and 12,532 NSS). Squares represent cohort-specific risk ratios (RRs), with size proportional to random-effects weight, and horizontal lines indicate 95% confidence intervals (CIs). The pooled random-effects estimate was RR = 1.10 (95% CI: 1.00–1.20; *p*=0.048), with moderate heterogeneity (I² = 59.7%; τ = 0.0900; *p* for heterogeneity = 0.011) and a 95% prediction interval of 0.87–1.38. The vertical solid line denotes the null effect (RR = 1), the dotted line the pooled estimate, the diamond the pooled RR and 95% CI, and the red bar the 95% prediction interval.

**FIGURE S6.** Leave-one-independent-cohort-out sensitivity analysis of the aggregated random-effects meta-analysis evaluating sanitary sheath (SS) use versus insemination without a sanitary sheath (NSS) for pregnancy per artificial insemination (P/AI) in cattle. Each row shows the pooled risk ratio (RR) after sequentially omitting one independent cohort. The primary random-effects estimate was RR = 1.07 (95% CI: 0.94–1.21; *p*=0.245), with a 95% prediction interval of 0.83–1.36. All leave-one-cohort-out point estimates remained above the null, ranging from RR = 1.04 to 1.10. However, all corresponding confidence intervals included 1.00, with *p*-values ranging from 0.084 to 0.342. Residual heterogeneity ranged from I² = 0% to 54.8% across iterations. Omission of Scariot et al. (2022) produced the greatest reduction in heterogeneity (I² = 0%; τ² = 0), indicating that this cohort contributed importantly to between-cohort variability.

**FIGURE S7.** Forest plots of exploratory subgroup analyses of sanitary-sheath (SS) use versus insemination without a sanitary sheath (NSS) for confirmed pregnancy per artificial insemination (P/AI) in cattle. A. Artificial-insemination method (estrus-detected AI vs TAI). B. Production system (beef vs dairy cattle). C. Protective-sheath type (flexible plastic bag-type cover vs rigid tube-type protective sheath). Points represent subgroup-specific pooled RRs, black horizontal lines indicate 95% confidence intervals, and red horizontal lines indicate 95% prediction intervals. The vertical line denotes the null effect (RR = 1). Differences between subgroup levels were evaluated using CR2 cluster-robust inference with Satterthwaite small-sample degrees of freedom.

**FIGURE S8.** Funnel-plot and small-study-effect sensitivity analyses for sanitary-sheath (SS) use and pregnancy per artificial insemination (P/AI) in cattle. **A.** Contour-enhanced funnel plot for the independence-restricted primary meta-analysis, including six independent cohorts. **B.** Trim-and-fill analysis of the primary independent dataset, showing the observed cohorts and one potentially missing comparison imputed on the left, together with the original and adjusted pooled estimates. In all panels, the x-axis represents the log risk ratio and the y-axis the corresponding standard error. Shaded regions indicate two-sided significance contours of *p* < 0.10, *p* < 0.05, and *p* < 0.01 relative to the null effect. Funnel-plot asymmetry and trim-and-fill analyses were considered exploratory because of the limited number of independent cohorts. **C.** Descriptive funnel plot after reintroducing the prespecified non-independent AI data; Schuenemann et al. (2011), representing an overlapping report, and Rezende et al. (2022), with unresolved data inconsistencies, are overlaid for transparency but were not included in this sensitivity model.

**FIGURE S9.** Influence diagnostics for the primary random-effects meta-analysis of sanitary-sheath (SS) use and pregnancy per artificial insemination (P/AI). **A.** Study-level influence diagnostics derived from the random-effects model, including externally studentized residuals (*rstudent*), DFFITS, Cook’s distance, covariance ratios, residual heterogeneity after study deletion (τ².del and QE.del), leverage values (*hat*), and random-effects weights. Horizontal reference lines indicate the diagnostic thresholds generated by the influence-analysis procedure. Scariot et al. (2022) had a studentized residual of 2.8149, exceeding the Bonferroni-adjusted threshold of 2.638, and was flagged as potentially influential. **B.** Baujat plot showing each study’s contribution to overall heterogeneity on the x-axis and its influence on the pooled estimate on the y-axis. Scariot et al. (2022) occupied the upper-right region, indicating the largest joint contribution to heterogeneity and influence. Study numbers correspond to: 1, Richards et al. (1984); 2, Kasimanickam (2016); 3, Silva et al. (2020); 4, Scariot et al. (2022); 5, Morales González (2023); and 6, Soytürk and Erdem (2024).
